# Abnormal Neutrophil Transcriptional Signature May Predict Newly Diagnosed Latent Autoimmune Diabetes in Adults of South China

**DOI:** 10.3389/fendo.2020.581902

**Published:** 2020-12-18

**Authors:** Yixuan Xing, Qiuqiu Lin, Yue Tong, Wenzhi Zhou, Juan Huang, Yanfei Wang, Gan Huang, Yanhua Li, Zhongyuan Xiang, Zhiguang Zhou, Tian Li, Yang Xiao

**Affiliations:** ^1^ National Clinical Research Center for Metabolic Diseases, and Department of Metabolism and Endocrinology, The Second Xiangya Hospital of Central South University, Changsha, China; ^2^ Department of Laboratory Medicine, The Second Xiangya Hospital of Central South University, Changsha, China; ^3^ School of Basic Medicine, Fourth Military Medical University, Xi’an, China

**Keywords:** latent autoimmune diabetes in adults, type 2 diabetes mellitus, neutrophils, RNA-seq, transcriptome, data mining

## Abstract

**Objective:**

Latent autoimmune diabetes in adults (LADA) is an autoimmune diabetes characterized by slowly progressive of β-cell function deterioration. Our previous finding demonstrated that neutrophil numbers and migration abilities display distinct levels in different types of diabetes, including LADA, whereas its pathological alterations in the development of LADA remain unknown. We aimed to investigate the changes in transcriptional levels of peripheral neutrophils in newly diagnosed LADA.

**Methods:**

Peripheral blood neutrophils were isolated from newly diagnosed LADA patients (n = 5) and age-and sex-matched healthy controls (n = 5). The Transcriptomic signature was determined by RNA sequencing (RNA-seq). Differentially expressed genes (DEG) were screened, followed by analyzing downstream Gene Ontology (GO) and Kyoto Encyclopedia of Genes and Genomes (KEGG) pathway enrichment. Real-time polymerase chain reaction (qPCR) was applied for validation in LADA patients (n = 9) and age-and sex-matched healthy controls (n = 18), including sequencing samples.

**Results:**

Compared with controls, 4105 DEG were screened in LADA patients, including 2661 upregulated and 1444 downregulated DEG. In GO analysis, DEG are mainly involved in leukocyte degranulation, myeloid cell differentiation, and immune response-regulating signaling. The top enriched KEGG pathways included cytokine-cytokine receptor interaction, adhesion molecule signaling, nuclear factor-κB (NF-κB) signaling and Th17 cell differentiation. Consistent with RNA-seq results, *SELL*, *ITGA4*, *ITGAM*, *NCF4*, *ARHGAP3*, and *CLDN15* are upregulated in neutrophils by qPCR.

**Conclusion:**

The present study results provided a profile of DEG in the newly diagnosed LADA of south China. Our study reveals an abnormality in neutrophil disposition at the transcriptional level in LADA. Several essential genes may be involved in of LADA’s pathological process, which may be useful to guide prediction for LADA and further investigation into the pathogenesis for this disease.

## Introduction

Diabetes mellitus is a global concern that causes an enormous burden to society and individuals. Latent autoimmune diabetes in adults (LADA) is a form of autoimmune diabetes with an older mean age at onset, slower rate of β-cell loss and longer period of insulin independence after onset when compared with type 1 diabetes (T1DM). In addition, early clinical manifestations overlap with those of type 2 diabetes (T2DM). Unlike T1DM, LADA does not initially require insulin for at least 6 months; however, deterioration of β-cell function in LADA patients is three times faster than that in T2DM (1). Global epidemiological surveys have indicated that LADA accounts for 2%–12% of patients with diabetes mellitus, who have more severe diabetic complications and a worse prognosis (2). Compared to T1DM, less intense autoimmune attack on β-cells and a relatively long window period from onset to β-cell depletion are usually found in LADA. This delayed progression period provides a valuable opportunity for endocrinologists to understand the pathological mechanisms of autoimmune destruction of β-cells.

Neutrophils are the most abundant white blood cells (WBCs) in the circulation (3) and are recruited to inflammatory sites (4) to eliminate extracellular pathogens after activation (5). NETosis, which is characterized by neutrophil degradation and successive release of lytic enzymes and neutrophil extracellular traps (NETs), is a unique process activated by neutrophils (6). However, aberrant activation of neutrophils during autoimmunity may aggravate inflammatory responses and tissue damage. Neutrophils also play a crucial role in several autoimmune diseases, including systemic lupus erythematosus (SLE), rheumatoid arthritis (RA), and autoimmune diabetes (3).

A longitudinal study from Battaglia’s group demonstrated that that neutrophil reduction is greatest in individuals with the highest risk of developing T1D, suggesting a closed correlation between reduced circulating neutrophils and destructive β-cell-specific autoimmunity in T1DM (7). Our group also showed a decrease in circulating neutrophil counts in patients with T1DM but not in those with T2DM (6), and additionally the circulating neutrophil counts in LADA is lower than those in T2DM and higher than those in T1DM (8). Moreover, levels of circulating protein and the activity of neutrophil serine protease 3 (PR3) and neutrophil elastase (NE) stored in primary neutrophil granules are significantly increased in T1DM (6) and LADA (9), and increased circulating levels of NE and PR3 exhibit a progressively positive correlation with the positive numbers and titres of islet autoantibodies (6). Recently, Battaglia et al. revealed an unexpected abnormal neutrophil signature both in the circulation and in the pancreas of presymptomatic and symptomatic T1DM subjects, implying that neutrophils might be involved in the pathogenesis of T1DM (10). However, the transcriptional profiling of neutrophils in LADA patients remains unclarified. In this study, we aimed to explore changes in peripheral neutrophils in the pathogenesis of LADA at the transcriptional level by comparing patients newly diagnosed LADA with healthy controls to provide a scientific basis for the screening of potential intervention targets.

## Materials and Methods

### Protocol

The study protocol, conforming to the Declaration of Helsinki (as revised in Seoul, South Korea, 2008), was reviewed and approved by the Human Ethics Committee of The Second Xiangya Hospital of Central South University (approval number 2019-Research-40). Bioinformatic data are acquired from public databases online.

### Subjects

Nine LADA patients diagnosed within one year were enrolled from the Second Hospital of Central South University, Changsha, China, and LADA were diagnosed according to the Chinese Diabetes Society (CDS) Consensus on diagnosis and treatment of LADA in 2012 (11): 1) diabetes diagnosed according to the 1999 World Health Organization (WHO) criteria for diabetes (12); 2) age at onset of diabetes of >18 years; 3) with one or more positive autoantibodies against β cell antigens including glutamic acid decarboxylase (GAD), insulinoma-associated protein 2 (IA2), or zinc transporter-8 (ZnT8); 4) insulin independence within the first six months after diagnosis. We recruited eighteen age-and sex-matched healthy controls who showed euglycemia in a standardized 75g oral glucose tolerance test (OGTT). The exclusion criteria were as follows: 1) acute infection, trauma, or surgery within one month; 2) treatments with glucocorticoids or other immune regulators within one month; 3) severe cardiocerebrovascular, liver, kidney, malignant disease; 4) pregnancy or lactation; 5) with other autoimmune diseases; 6) other types of diabetes (13–17). Five LADA patients and five controls were selected *via* random number table method for RNA-sequencing (RNA-seq), and all subjects were used as validation samples for Real-time polymerase chain reaction (qPCR).

### Measurements

Height, weight, blood pressure, waist circumference, hip circumference, body mass index (BMI), and weight/height ratio (WHR) were calculated for all participants. Fasting venous blood samples were collected at 8:00 am. Biomedical measurements were tested for serum or plasma separation upon blood collection. Fasting blood glucose (FBG), total cholesterol (TC), and triglyceride (TG) were measured by a Hitachi 7170 analyzer (Boehringer Mannheim, Mannheim, Germany). Circulating cell counts were measured by the Sysmex XE-2100 automated hematology analyzer (Sysmex Corporation, Kobe, Japan). Serum C-peptide was measured by the Advia Centaur System (Siemens Corporation, Munich, Germany). Glycosylated hemoglobin (HbA1c) was quantified by liquid chromatography using a Bio-Rad VARIANT II Hemoglobin Testing System (Hercules, CA, USA). Glutamic acid decarboxylase autoantibody (GADA), insulinoma-associated protein 2 autoantibody (IA-2A), and zinc transporter-8 autoantibody (ZnT8A) were detected in duplicate by radio ligand assays as previously described (18, 19).

### Neutrophil Isolation and RNA Extraction

According to the manufacturer, human neutrophils were isolated by density gradient centrifugation from the venous blood of both LADA patients and healthy controls using Ficoll-Paque Plus (GE Healthcare, Madison, USA)’s protocol. Further purification was completed by positive magnetic separation using human CD16 Microbeads (Miltenyi Biotec, Bergisch Gladbach, Germany). The cell pellet was dissolved in TRIzol (Roche, Basel, Switzerland) in 5–10 × 10^6 cells/1 ml and stored at -80°C. Total RNA was extracted, and corresponding concentration and purity were evaluated on a NanoDrop spectrophotometer (Thermo Fisher Scientific, Waltham, MA, USA). Standard OD260/OD280 value of extracted RNA is 1.8–2.1. The High-Capacity cDNA Reverse Transcription Kit (Thermo Fisher Scientific, Waltham, MA, USA) subsequently constructed the cDNA library.

### RNA Sequencing

In this study, we sequenced five samples from LADA patients and five samples from healthy controls on the BGISEQ-500 platform, the first desktop high-throughput gene sequencer independently developed by BGI, and applies DNA nanoball technology. A total of 15,255 genes were detected, averaging approximately 24.04 million reads per sample.

### Data Cleaning and Bio Information Analysis Obtained From the Public Database

We carried out quality control of the raw data before downstream analysis, and filter out the clean reads through the steps of low quality, adapter pollution and unknow base (N) reads. Then, clean reads were mapped to the reference genome using HISAT (20) and Bowtie2 (21), and the gene expression calculated using a software package called RSEM (University of Wisconsin-Madison, Madison, USA) (22). We identified differentially expressed genes (DEG) between LADA patients and healthy controls by DEG-seq algorithms (23). An adjusted P-value ≤ 0.001 and an absolute value of the log_2_ fold change (FC) > 1 were set as the default threshold to judge the significance of gene expression differences. According to DEG, we used Metascape online website to perform GO functional enrichment, including three ontologies: molecular biological function, cellular composition, and biological process (24). Further, we performed the Kyoto Encyclopedia of Genes and Genomes (KEGG) pathway functional enrichment, using hyper package in R software. Then we calculated the false discovery rate (FDR) for each P-value, and the terms for which the FDR was not greater than 0.01 were defined as significantly enriched.

### Real-Time PCR Analysis

To validate the accuracy of the RNA-seq analysis, the expression of mRNAs was measured by real-time polymerase chain reaction (qPCR) using the SYBR-Green method (Go Taq^®^ qPCR, Promega Corporation, USA) and a MiniOpticon real-time PCR detection system (ViiATM 7 Real-Time PCR System containing the OptiflexTM Optics System). All the primers used for real-time PCR were designed and synthesized by TSINGKE (TSINGKE Biological Technology, China). Real-time PCR reactions were performed under the following conditions: 10 min at 95°C and 40 cycles of the one-step thermal cycling of 15 s at 95°C and 60 s at 60°C in a 384-well reaction plate. The expression of each gene was determined in duplicates based on the basis of the comparative 2^-ΔΔCt^ method. Results were normalized to the expression of reference gene β-actin. Primer sequences of genes were shown in **Table 1**.

**Table 1:** Primer sequences of forward and reverse primers.

Gene	Sense (5’ to 3’)	Antisense (3’ to 5’)
**CXCR1**	TCAAGTGCCCTCTAGCTGTT	TGATCTAACTGAAGCACCGGC
**SELL**	TCTGTTGTGATTTCCTGGCAC	CCCACCCACGTCCATATTCC
**ITGA4**	CGGTGATGCTGTTGCTGTG	CTAGGAGCCATCGGTTCGCC
**ITGAM**	GGTGGCAGTGTGATGCTGT	CATTTACGTCCCCCAGCACT
**NCF4**	GGCTGGAGGAAGTGAGAGGT	TGTTCAAAGTCACTCTCGGC
**ARHGAP35**	TAAACAAGGTCAGCCACAACA	TCAGGTCTCATCAAGGTGGG
**CLDN15**	TTACTCCGTTCTTTGGCCCC	GCGCGGCAGCCTGGA
**β-Actin**	GCATCCCCCAAAGTTCACAA	AGGACTGGGCCATTCTCCTT

### Statistical Analysis

All data were analyzed with SPSS version 25 (IBM Corp, Armonk, NY, USA) and GraphPad Prism 5 (Graphpad Corporation, San Diego, CA, USA). Normality was tested using the Kolmogorov-Smirnov test. Data were logarithmically transformed before the Mann-Whitney U test if they were not normally distributed. Student’s t-test was applied to identify differences between groups. Univariate general linear model was used to exclude the potential influence of confounding factors, regarded as covariates. Data are expressed as the mean ± SD or median with interquartile range.

## Results

The clinical and metabolic characteristics of LADA patients (n=9) and age- and sex-matched control subjects (n=18) are presented in **Tables 2** and **3**. Five patients with LADA and five healthy controls were randomly selected for RNA-seq, and qPCR validation was applied to the whole group. Levels of HbA1c (P < 0.05), FBG (P < 0.01), and 2-h postprandial blood glucose (P < 0.05) were higher in LADA patients than in healthy controls. In the validation group, the 2-h postprandial C-peptide in patients with LADA was lower than that in healthy controls (P < 0.001). However, no statistical significance in BMI, systolic blood pressure, LDL-C, or TC was found between the LADA and control subjects (P>0.05).

**Table 2:** Clinical and biochemical characteristics of the study participants for RNA-seq.

	HC (n=5)	LADA (n=5)	P
**Sex (male/female)**	5 (4/1)	5 (3/2)	0.490
**Age (years)**	43.40 ± 13.22	34.4 ± 4.98	0.212
**BMI (kg/m²)**	23.18 ± 2.21	22.95 ± 2.21	0.872
**WHR**	0.89 (0.80 ~ 0.91)	0.81 (0.80~0.87)	0.841
**DBP (mmHg)**	74.40 ± 5.32	71.8 ± 5.12	0.860
**SBP (mmHg)**	106.20 ± 8.95	107.8 ± 6.94	0.760
**TG (mmol/L)**	1.03 ± 0.56	1.25 ± 0.68	0.588
**TC (mmol/L)**	4.22 ± 0.49	3.82 ± 0.56	0.263
**HDL-C (mmol/L)**	1.47 ± 0.44	1.29 ± 0.31	0.480
**LDL-C (mmol/L)**	2.34 ± 0.54	2.1 ± 0.6	0.522
**HbA1C (%)**	5.52 ± 0.46	6.62 ± 0.89*	0.039
**Fasting BS (mmol/L)**	5.12 ± 0.29	6.27 ± 0.58**	0.004
**2h postprandial BS (mmol/L)**	4.88 ± 1.64	12.05 ± 4.88*	0.027
**Fasting C-peptide (pmol/L)**	350.36 ± 90.08	290.34 ± 80.39	0.299
**2h postprandial C-peptide (pmol/L) a**	1672.8(1117.7~1885.15)	654.8(503.1~1033.1)	0.095
**White cell count (10^9/L) a**	6.34(5.27~7.50)	4.74(4.24~6.19)	0.150
**Lymphocyte count (10^9/L) a**	1.83(1.38~2.20)	1.44(1.24~1.89)	0.363
**Neutrophil count (10^9/L) a**	3.45 (3.25~5.40)	2.88(2.47~3.92)	0.190
**Mononuclear count (10^9/L) a**	0.27(0.22~0.46)	0.25(0.22~0.38)	0.608

Data are expressed by mean ± SD, or median (25th–75th percentile). LADA, Latent autoimmune diabetes in adults; HC, Healthy controls; BMI, Body mass; WHR, Waist to hip ratio; DBP, diastolic blood pressure; SBP, systolic blood pressure; TG, Triglycerides; HDL, high-density lipoprotein; LDL, low-density lipoprotein; a compared by the Mann-Whitney U test. *P < 0.05 compared with HC. **P < 0.01 compared with HC.

**Table 3:** Clinical and biochemical characteristics of the study participants for validation.

	HC (n = 18)	LADA (n = 9)	P	HC (N = 5)	T2DM (N = 5)	P
**Sex (male/female)**	18 (8/10)	9 (5/4)	0.586	5(3/2)	5(3/2)	1.000
**Age (years) **	33.94 ± 10.34	34.56 ± 6	0.891	32.60 ± 5.94	50.8 ± 8.98**	0.005
**BMI (kg/m²)**	21.59 ± 1.9	21.87 ± 2.94	0.804	23.26 ± 2.32	23.54 ± 5.61	0.919
**WHR**	0.83 (0.80~0.89)	0.81 (0.81~0.87)	0.743	0.90 ± 0.05	0.91 ± 0.06	0.849
**DBP (mmhg)**	72.33 ± 6.91	71 ± 5.57	0.620	77.20 ± 11.78	78.60 ± 14.22	0.870
**SBP (mmhg)**	108.94 ± 9.53	109 ± 7.53	0.988	123.80 ± 14.87	127.00 ± 28.36	0.829
**TG (mmol/l)**	0.93 ± 0.41	0.98 ± 0.61	0.804	1.67 ± 1.09	2.61 ± 1.28	0.244
**TC (mmol/l)**	3.99 ± 0.54	3.84 ± 0.77	0.572	5.41 ± 0.81	5.50 ± 0.71	0.853
**HDL-C (mmol/l)**	1.41 ± 0.29	1.3 ± 0.26	0.372	1.34 ± 0.26	1.22 ± 0.12	0.351
**LDL-C (mmol/l)**	2.35 ± 0.86	2.13 ± 0.66	0.513	3.71(2.86~3.89)	3.22(2.64~3.40)	0.151
**HbA1c (%)**	5.31 ± 0.33	6.67 ± 1.33*	0.015	5.36 ± 0.29	9.34 ± 2.58*	0.026
**Fasting bs (mmol/l)**	4.7 ± 0.49	5.89 ± 0.89***	<0.001	4.85 ± 0.53	12.79 ± 5.51*	0.032
**2h postprandial bs (mmol/l)**	5 ± 1.13	10.38 ± 4.38**	0.006	7.59 ± 1.83	16.16 ± 3.14***	<0.001
**Fasting c-peptide (pmol/l) **	345.98 ± 96.51	275.20 ± 64.06	0.058	1067.60 ± 402.76	599.40 ± 335.93	0.081
**2h postprandial c-peptide(pmol/l)**	1687.25 ± 657.2	654.80 (590.40~895.15) ***	<0.001	3779.55 ± 1018.38	2217.78 ± 1675.14	0.113
**White cell count (10^9/l) a**	5.83 (4.90~6.38)	4.68 (4.39~6.52)	0.180	6.42 ± 1.31	5.36 ± 1.14	0.211
**Lymphocyte count (10^9/l) a**	1.67 (1.21~1.93)	1.43 (1.22~1.87)	0.9112	1.88(1.80~2.65)	1.86(1.44~3.28)	1.000
**Neutrophil count (10^9/l) a**	3.58 (3.27~4.18)	2.86 (2.61~4.37)	0.090	3.78 ± 1.07	2.80 ± 0.21	0.080
**Mononuclear count (10^9/l) a**	0.30 (0.25~0.45)	0.25 (0.20~0.33)	0.160	0.37 ± 0.08	0.28 ± 0.08	0.123

The study participants for validation included the testing samples for RNA-seq. Data are expressed by mean ± SD, or median (25th-75th percentile). LADA, Latent autoimmune diabetes in adults; T2DM, Type 2 diabetes mellitus; HC, Healthy controls; BMI, Body mass; WHR, Waist to hip ratio; DBP, diastolic blood pressure; SBP, systolic blood pressure; TG, Triglycerides; HDL, high-density lipoprotein; LDL, low-density lipoprotein; a compared by the Mann-Whitney U test. *P< 0.05 compared with HC. ** P< 0.01 compared with HC. *** P <0.001 compared with HC.

**Figure 1:**
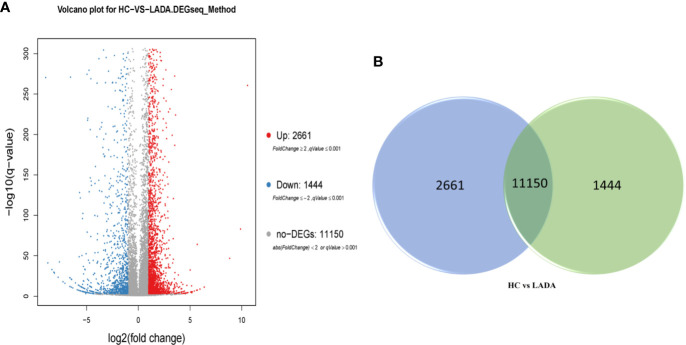
**(A)** Volcano plot of differentially expressed genes (DEG) in peripheral neutrophils obtained from newly diagnosed LADA patients vs. healthy controls. Red points represent up-regulated DEG. Blue points represent down-regulated DEG. Gray points represent non-DEG. A total of 2,661 upregulated DEG and 1,444 downregulated DEG were found. The X-axis represents log2 transformed fold change, and Y-axis represents -log10 transformed significance. The BGISEQ-500 platform was used to compare to the reference sequence to screen the differential genes. **(B)** Veen diagram of differentially expressed genes (DEG). The numbers of DEG in peripheral neutrophil obtained from newly diagnosed LADA patients vs healthy controls. The blue pie represents 2,661 up-regulated DEG. The green pie represents 1,444 down-regulated DEG. The transposition section represents 11150 non-DEG.

**Figure 2:**
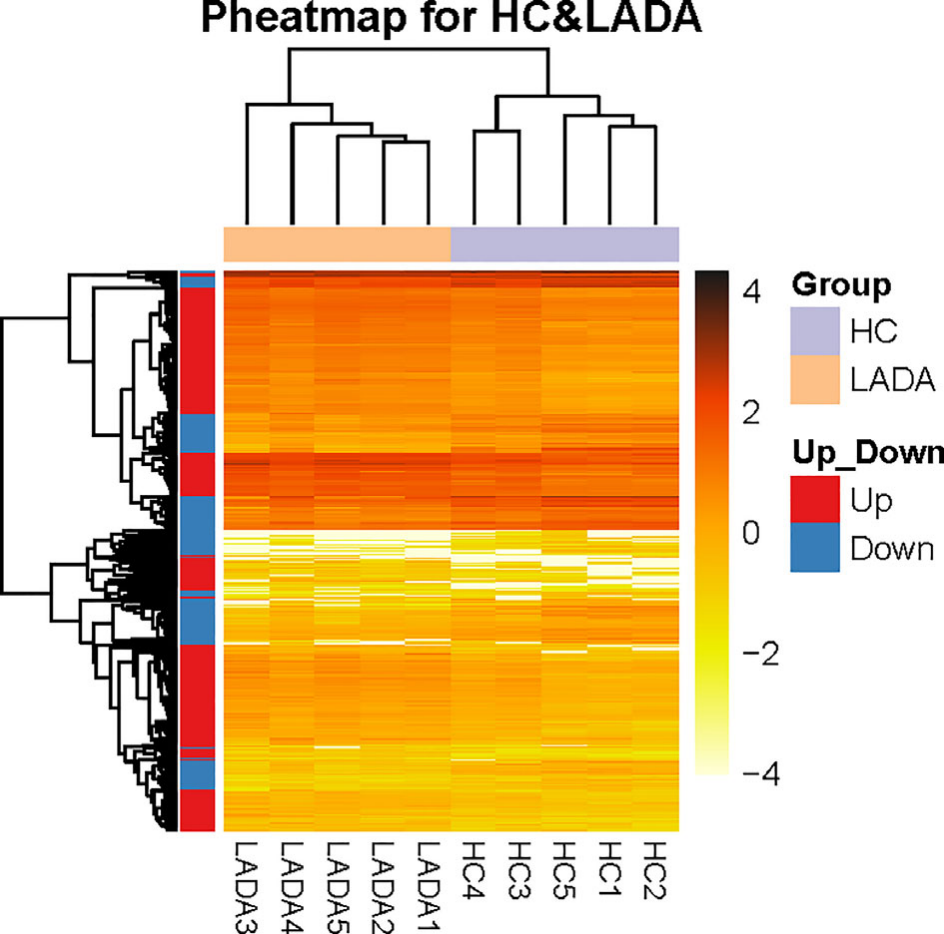
The distinct peripheral neutrophil transcriptome of patients with newly diagnosed latent autoimmune diabetes in adults (LADA) vs healthy controls. Heatmap shows diﬀerentially expressed genes (DEG) in neutrophils isolated from healthy controls (purple, n = 5) and patients with newly diagnosed LADA (orange, n = 5). DEGs are defined as levels of fold change ≥ 2 for up-regulated and ≤ 0.5 for down-regulated. The X-axis indicated different LADA patients and healthy controls, and the Y-axis shows DEG. The orange color scale bar represents the log10 transformed gene expression level; dark orange represents high expression level, and light orange represents low expression.

RNA-seq analysis of peripheral blood neutrophils from 5 patients with LADA and 5 healthy controls revealed an average of 24.04 million sequences in each sample, with a reading mapping rate of 93.24% (See **Supplementary Table 1** and **2**). According to the DEG-seq algorithm, among the 15,255 transcripts that could detect differences in expression, 2,661 DEG were upregulated and 1,444 DEG downregulated in patients with LADA compared with healthy controls. The DEG were defined at levels of FC ≥ 2 or fold change ≤ 0.5 and adjusted P-value ≤ 0.001 (**Figures 1**, **2**, and **Supplementary Data 1**).

**Figure 3:**
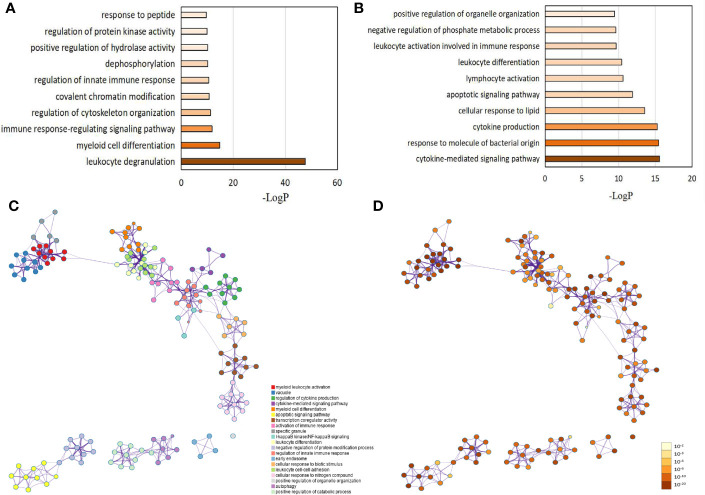
Gene ontology (GO) analysis for differentially expressed genes (DEG) between LADA patients and healthy controls. DEGs are defined as levels of fold change ≥ 2 for upregulated and ≤ 0.5 for downregulated. GO analysis is carried out using more than 40 independent knowledge bases used by the Metascape online website. **(A)** The top 10 GO categories for up-regulated genes. **(B)** The top 10 GO categories for down-regulated genes. The network of enriched GO terms****: **(C)** Colored by the term, where nodes that share the same term are typically close to each other; **(D)** Colored by p-value, where terms containing more genes tend to have a more significant p-value. Each term is represented by a circle node, where its size is proportional to the number of input genes that fall into term, and its color represent its cluster identity. Terms with a similarity score > 0.3 are linked by an edge (the edge’s thickness represents the similarity score). The darker the color, the greater the statistical significance of the node. The GO network is also analyzed using the Metascape online website.

Based on the aforementioned DEG, GO classification and functional enrichment analyses were performed to determine the molecular functions (MM), cellular components (CC), and biological processes (BP) involving the proteins encoded by these genes. Among the upregulated and downregulated genes, we selected the first 1,000 DEGs as the background. Terms with a p-value < 0.01, a minimum count of 3, and an enrichment factor > 1.5 were collected and grouped into clusters based on similarities. As expected, most identified pathways are important to neutrophil functioning. Among them, the top 10 upregulated biological processes were as follows: 1) leukocyte degranulation (Log P = -43.90); 2) myeloid cell differentiation (Log P = -14.74); 3) immune response-regulating signaling (Log P = -11.80); 4) regulation of cytoskeleton organization (Log P = -11.22); 5) covalent chromatin modification (Log P = -10.72); 6) regulation of innate immune response (Log P = -10.54; 7) dephosphorylation (Log P = -10.20; 8) positive regulation of hydrolase activity (Log P = -10.08); 9) regulation of protein kinase activity and response to peptide (Log P = -9.86); and 10) response to peptide (Log P = -9.656) (**Figure 3A** and **Supplementary Data 2**). The corresponding GO categories of the top 10 upregulated biological functions were 1) cytokine-mediated signaling pathway (Log P = -15.55); 2) response to molecule of bacterial origin (Log P =-15.43); 3) cytokine production (Log P = -15.26); 4) cellular response to lipid (Log P = -13.56); 5) apoptotic signaling pathway (Log P = -11.88); 6) lymphocyte activation (Log P = -10.61); 7) leukocyte differentiation (Log P = -10.39); 8) leukocyte activation involved in immune response (Log P = -9.65); 9) negative regulation of phosphate metabolic process (Log P = -9.62); and 10) positive regulation of organelle organization (Log P = -9.41) (**Figure 3B** and **Supplementary Data 2**). At the same time, we generated a network diagram of GO terms with significant enrichment; the network was visualized using Cytoscape5, where each node represents an enriched term and is coloured first by its term (**Figure 3C**) and then by its P-value (**Figure 3D**).

**Figure 4:**
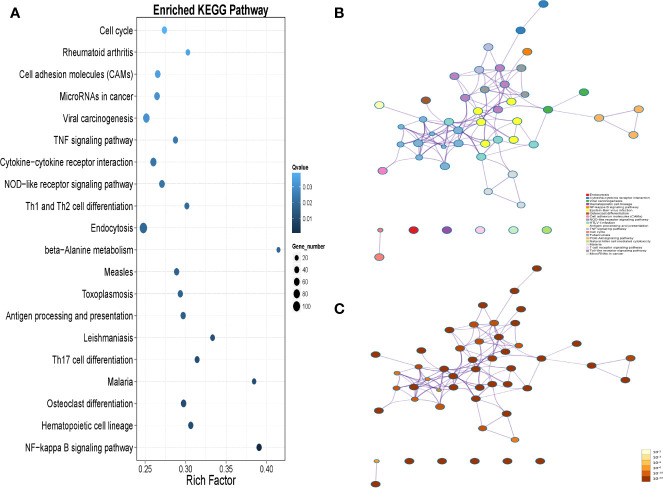
**(A)** The top 20 Kyoto Encyclopedia of Genes and Genomes (KEGG) pathways are based on all the differentially expressed genes (DEGs) in peripheral neutrophils between latent autoimmune diabetes in adults (LADA) patients and healthy controls. DEG is defined as levels of fold change ≥ 2 for up-regulated and ≤ 0.5 for down-regulated. The enrichment results of the KEGG pathway are obtained from the KEGG database. The X-axis represents enrichment factors, and the Y-axis represents pathway names. The blue color scale bar indicates the q-value (high: light, low: dark), and the lower Q-value indicates the more significant enrichment. Point size indicates DEG numbers (bigger dots refer to more massive amounts). Rich Factor refers to the enrichment factor’s value, which is the quotient of foreground value (the number of DEG) and background value (total Gene amount). The larger the value, the more significant enrichment. **(B)** KEGG-DEG relationship network colored by the pathway, where nodes that share the same pathway are typically close to each other; **(C)** KEGG-DEG relationship network colored by p-value, where pathway containing more genes tend to have a more significant p-value.

**Figure 5:**
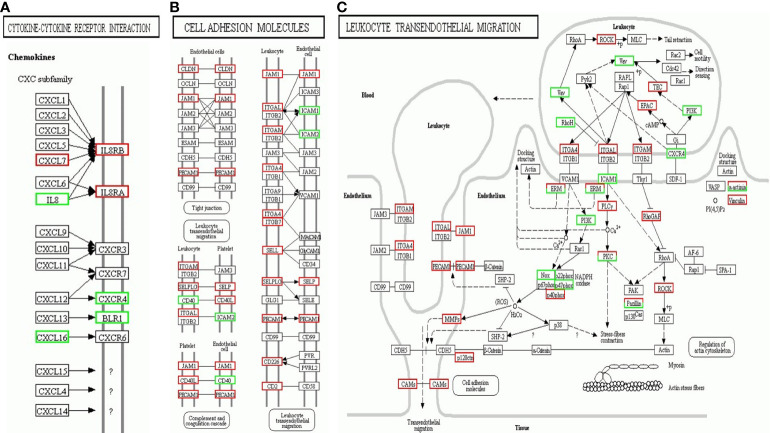
Kyoto Encyclopedia of Genes and Genomes (KEGG) Pathway map enriched by differentially expressed genes (DEG) of neutrophils from latent autoimmune diabetes in adults (LADA) patients compared with healthy controls. **(A)** Cytokine-cytokine receptor interaction; **(B)** Cell adhesion molecules; **(C)** Leukocyte transendothelial migration. Red represents upregulated genes, and the green represents downregulated genes.

KEGG pathway enrichment analysis of the DEG suggested that a wide range of biological pathways are altered in neutrophils from LADA patients compared to controls. In KEGG pathway enrichment analysis, a total of 321 pathways were identified based on all detected genetic backgrounds. The top 20 differential pathways are mainly related to the NF-κB signaling pathway (P = 2.56×10^-8^), Th17 cell differentiation (P = 1.75×10^-4^), antigen processing and presentation (P = 3.54×10^-4^), Th1 and Th2 cell differentiation (P = 7.40×10^-4^), NOD-like receptor signaling pathway (P = 9.91×10^-4^), Cytokine-cytokine receptor interaction (P=1.00×10^-3^), TNF signaling pathway (P = 1.13×10^-3^), and Cell adhesion molecules (CAMs) (P = 1.96×10^-3^). (**Figure 4A** and **Supplementary Data 3**, Q value in **Supplementary Table 3** and in the text are adjusted P-value). We drew a network map between the top 20 pathways with the highest enrichment degree and the corresponding DEG (**Figures 4B, C**). Among the most enriched pathways mentioned above, especially cytokine-cytokine receptor interactions and CAM signal transduction pathways, most of the genes related to these two pathways were found to be upregulated in LADA, such as chemokine (C-X-C subfamily) ligand (CXCL7) and C-X-C chemokine receptor type (CXCR1), also known as interleukin-8 receptor (IL8RA, IL8RB), cell adhesion molecules L-selectin (*SELL*) and integrin subunit alpha M (*ITGAM*). The paths of cytokine-cytokine receptor interaction, cell adhesion molecules and leukocyte transendothelial migration are illustrated in **Figure 5**.

**Figure 6:**
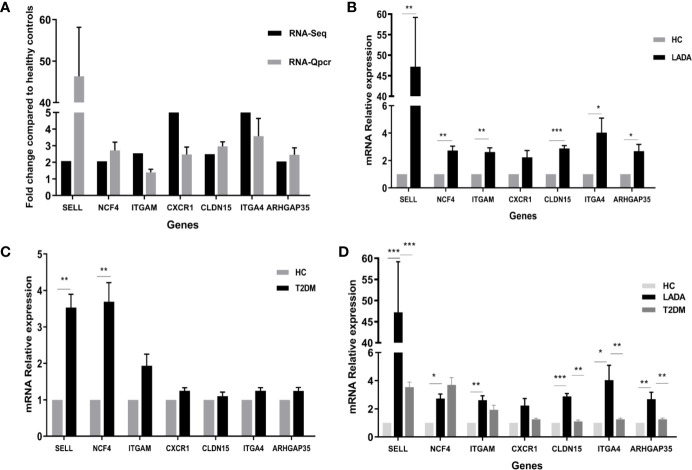
qPCR validation for genes identified by RNA-Seq. **(A)** Black bars denote the RNA-Seq fold change values of genes identified by RNA-seq compared to healthy controls, while gray bars represent real-time polymerase chain reaction (qPCR) fold change values calculated using the 2^^-ΔCt^ method. The 2^^-ΔCt^ of gene expression from latent autoimmune diabetes in adults (LADA) patients and healthy controls were analyzed, and then the fold change were calculated by 2^^-ΔCt^ of gene expression from LADA patients divided by 2^^-ΔCt^ of gene expression from controls. Data are presented as the mean ± SD. **(B)** Expression of seven genes in neutrophils from patients with LADA and healthy controls (HC). The ordinate represents the relative mRNA expression, calculated using the 2^^-ΔΔCt^ method. The black bar represents the gene expression in neutrophils from LADA, while the gray bar represents the gene expression in neutrophils from healthy control (HC). *P < 0.05, **P < 0.01, ***P < 0.001. **(C)** Expression of seven genes in neutrophils from patients with type 2 diabetes (T2DM) and HC. The ordinate represents the relative mRNA expression, calculated using the 2^^-ΔΔCt^ method. The black bar represents the gene expression in neutrophils from T2DM, while the gray bar represents the gene expression in neutrophils from HC. *P < 0.05, **P < 0.01, ***P < 0.001. **(D)** Expression of seven genes in neutrophils from all HC, LADA and T2DM. The ordinate represents the relative mRNA expression, calculated using the 2^^-ΔΔCt^ method. The black bar, dark gray bar and light gray bar represents the gene expression in neutrophils from LADA, T2DM, and HC, respectively. *P < 0.05, **P < 0.01, ***P < 0.001.

We next confirmed the differential expression of 7 prominent DEG closely related to neutrophil activation, rolling and migration using qPCR. These verified genes showed the same direction as in the RNA-seq data (**Figure 6A**). The expression levels of adhesion molecules *SELL, ITGAM, ITGA4*, and neutrophil cytoplasmic factor *NCF4, ARHGAP35, CLDN15* were significantly increased in neutrophils from LADA patients compared with those from controls, except for *CXCR1* (**Figure 6B**). We further measured and compared the gene expression of *CXCR1, SELL, ITGA4, ITGAM, NCF4, ARHGAP3*, and *CLDN15* in neutrophils from 5 type 2 diabetes patients within 1 year from diagnosis and 5 age- and sex-matched healthy control subjects. There was no significant difference in gene expression of *CXCR1, ITGA4, ITGAM, ARHGAP3*, and *CLDN15* between the two groups (**Figure 6C**). The relative mRNA expression of above genes was also compared among all healthy controls, LADA and T2DM (**Figure 6D**). To further exclude the potential confounder of hyperglycemia, HbA1c was adjusted by univariate general linear model, and the expression of *SELL, ITGA4, ITGAM, NCF4, ARHGAP3*, and *CLDN15* remained significantly higher in neutrophils from LADA, but not T2DM, compared with healthy controls; and the expression of *SELL, ITGA4, ARHGAP3*, and *CLDN15* remained significantly higher in neutrophils from LADA compared with those from T2DM. Taken together, these data suggest that *SELL, ITGA4, ARHGAP35, CLDN15* may be closely associated with β-cell autoimmunity in patients with LADA, and the increase of other genes in LADA may only reflect the hyperglycemia status.

## Discussion

In this study, the transcriptional signature of circulating neutrophils in newly diagnosed LADA patients was found to be different from that of healthy controls without diabetes, suggesting that neutrophils may play a role in the pathogenesis of human autoimmune diabetes. In addition, levels of *SELL, NCF4, ITGAM, ITGA4, ARHGAP35*, and *CLDN* were significantly increased in neutrophils from LADA patients compared to those from healthy controls.

Emerging evidence have demonstrated the role of neutrophils involved in autoimmune diabetes. Battaglia et al. have reported reduction of circulating neutrophil numbers and infiltration of neutrophils in the pancreas before the onset of T1DM, suggesting that pathogenic role of neutrophils in human T1D is crucial for a better understanding of the disease and to open new therapeutic opportunities (7, 25). Our previous findings have demonstrated that in newly diagnosed T1DM patients, a significantly decreased number of circulating neutrophils and increased circulating neutrophil PR3 and NE levels are related to decreased β-cell function and the number and titres of islet autoantibodies (6). The level of circulating PR3 was also significantly increased in patients with LADA, which is considered a milder form of T1DM. In this study, we prove that the transcriptional signatures of circulating neutrophils change significantly in the early stage of LADA.

In the current study, DEG and related biological functions were investigated in peripheral neutrophils of patients with LADA and healthy controls. The results showed that pathways involving migration, adhesion, and degranulation were activated in circulating neutrophils at the transcriptional level in LADA. Increasing evidence has demonstrated that in autoimmune diabetes, the migration of neutrophils from circulation to islets is involved in the early stage of β-cell injury (3). Circulating neutrophil counts have been observed to decrease in presymptomatic autoantibody-positive donors and T1DM patients, and neutrophil infiltration was also found in human islet sections (10). Recruitment of neutrophils also occurred in 3-week-old nonobese diabetic (NOD) mice (26). The first step of local tissue infiltration of neutrophils is passage through vascular endothelial cells. We found that a series of adhesion molecules were upregulated in the neutrophils of LADA patients. Adhesion molecules not only play essential roles in the ability of neutrophils to pass through endothelial cells into local tissue but also participate in regulation of the immune system (3). Our RNA-seq and qPCR results both showed that *SELL*, the gene encoding the adhesion molecule L-selectin, was significantly increased in neutrophils from LADA patients. A previous study found that soluble L-selectin (sL-selectin) was altered in patients with T1DM and preclinical T1DM (27). Subsequent studies have shown that increased expression of sL-selectin may promote the destructive process of islet inflammation during the development of T1DM (28, 29). In addition, elevation of sL-selectin has been related to the positive serum conversion of islet autoantibodies, indicating that the activation of leukocytes is consistent with the occurrence of β-cell autoimmunity (30). Moreover, our results showed that *ITGA4* and *ITGAM*, which encode very late antigen 4 (VLA4) and CD11b, respectively, were significantly upregulated. Antibodies against L-selectin and VLA-4 can delay insulitis by inhibiting leukocyte adhesion to the inflamed vessels within pancreatic sections (31). Neutrophils of T1DM patients showed higher expression of the CD11b receptor than that of healthy controls, independent of diabetes duration and blood glucose level (32). These studies have shown that adhesion molecules, especially L-selectin and VLA-4, play significant roles in the development of T1DM, and the development can be halted by blocking these adhesion pathways. Therefore, further studies are required to elucidate the preventive effects of neutrophil-specific anti-adhesion therapies in the progression of LADA.

Our GO analysis showed that leukocyte degranulation was the most conspicuous biological process. Consistent with our previous results indicating increased serum PR3 in LADA patients, neutrophil degranulation is accompanied by the release of serine proteases in azurophilic granules, including PR3 and NE (33, 34), and an increase in PR3 gene expression (35). During the degranulation of neutrophils, neutrophil-induced ROS production is increased in both patients and rat models of T1DM (36, 37). These toxic substances not only damage pancreatic β-cells but also destroy the antioxidant defence of neutrophils (38–40). However, the specific molecular mechanism by which NADPH is activated in autoimmune diabetes remains unknown. According to our results, expression of *NCF4* and *ARHGAP35* in patients with LADA was upregulated compared with in healthy controls, and qPCR validated this result. The protein P40phox encoded by *NCF4* is the cytoplasmic regulator of NADPH oxidase in superoxide phagocytes; and *p190RhoGAP* encoded by *ARHGAP35* can also increase NADPH oxidase by inhibiting intracellular rac1. Thus, we speculate that neutrophils increase utilization of NADPH and the level of reactive oxygen species (ROS) in LADA through upregulation of *NCF4* and *ARHGAP35*, contributing to direct damage to β-cells. Overall, further research is needed to determine whether *NCF4* and *ARHGAP35* mediate ROS damage to β-cells *via* neutrophils and whether they can be used as intervention targets.

This study has potential limitations. First, the sample size was relatively small, and only newly diagnosed cases from a single center were included. Larger sample sizes and multicenter research need to be carried out. Second, due to the lack of deep basic research, we were unable to elucidate the precise role of the key genes we identified in LADA. Thus, it is necessary to further explore the specific molecular mechanisms of these genes in the pathogenesis of LADA. Third, the cross-sectional design cannot suggest a causal relationship between neutrophils abnormalities and the development of LADA, thus the role of neutrophils in LADA remains to be confirmed by larger long-term follow-up studies.

## Conclusion

Our study firstly investigated the DEG in circulating neutrophils of LADA patients as well as corresponding biological functions. In patients with LADA, neutrophils showed activation of degranulation, adhesion, and migration at the transcriptional level. Four essential genes, such as *SELL, ITGA4, ARHGAP35*, and *CLDN15*, may be involved in the pathological process of LADA. These results suggest that neutrophilic dysfunction may play a role in LADA’s pathological process, providing a possible prospect for predicting the onset of LADA and exploring the pathogenesis of LADA.

## Data Availability Statement

The datasets presented in this study can be found in online repositories. The datasets are deposited to Figshare and available at: https://figshare.com/articles/dataset/Abnormal_neutrophil_transcriptional_signature_may_predict_newly_diagnosed_latent_autoimmune_diabetes_in_adults_of_South_China/12651740.

## Ethics Statement

The studies involving human participants were reviewed and approved by Human Ethics Committee of The Second Xiangya Hospital of Central South University. The patients/participants provided their written informed consent to participate in this study.

## Author Contributions

YXX wrote the manuscript. QL, WZ, JH, YL, and ZX collected the samples. YXX, QL, and YT performed the experiment and analyzed the data. YX, YT, and TL revised the manuscript. YX, ZZ, and GH designed the study. All authors contributed to the article and approved the submitted version.

## Funding

This work was supported by the National Science Foundation of Hunan Province for Excellent Young Scholars (2020JJ3056 to YX), the National Natural Science Foundation of China (NSFC) grant (81670772, 81870577 to YX), the National Key Research and Development Project (2018YFE0114500 to YX, 2016YFC1305000, 2016YFC1305001 to ZZ, and 2018YFC1315603 to GH), and the Science and Technology Major Project of Hunan Province (2017SK1020 to ZZ).

## Conflict of Interest

The authors declare that the research was conducted in the absence of any commercial or financial relationships that could be construed as a potential conflict of interest.
