# Supplementary material for: Abnormal Neutrophil Transcriptional Signature May Predict Newly Diagnosed Latent Autoimmune Diabetes in Adults of South China
Source: Front Endocrinol (Lausanne). 2020 Dec 18;11:581902. doi: 10.3389/fendo.2020.581902 (PMC7775642; doi:10.3389/fendo.2020.581902)
Supplement: Supplementary file 2 [file Table_1.docx]

[Supplementary](https://www.sciencedirect.com/science/article/pii/S0378111919308182?via=ihub#s0110) Table 1 RNA-Seq sequences reads mapping to reference genome using HISAT

| Sample | Total Clean Reads | | Total Mapping Ratio (%) | Uniquely Mapping Ratio (%) |
| --- | --- | --- | --- | --- |
| HC1 | | 23988514 | 93.97 | 82.23 |
| HC2 | | 23981056 | 93.48 | 81.64 |
| HC3 | | 24030172 | 93.18 | 81.76 |
| HC4 | | 23988688 | 93.54 | 81.33 |
| HC5 | | 24022970 | 93.29 | 81.25 |
| LADA1 | | 24084976 | 92.81 | 81.30 |
| LADA2 | | 24065044 | 92.86 | 81.20 |
| LADA3 | | 24101688 | 93.49 | 81.84 |
| LADA4 | | 24066643 | 92.63 | 80.67 |
| LADA5 | | 24081934 | 93.13 | 81.64 |

HC: heathy control; LADA: Latent autoimmune diabetes in adults；Total Clean Reads: The amount of Clean reads；Total Mapping Ratio: The percentage of mapped reads (%)；Uniquely Mapping Ratio: The percentage of uniquely mapped reads (%).

[Supplementary](https://www.sciencedirect.com/science/article/pii/S0378111919308182?via=ihub#s0110) table 2 RNA-Seq sequences reads mapping to reference transcripts using Bowtie2

| Sample | Number of clean reads (mb) | Number of mapped reads (mb) | Mapped reads (%) |
| --- | --- | --- | --- |
| HC1 | 23988514 | 14795177 | 61.68 |
| HC2 | 23981056 | 14144440 | 58.98 |
| HC3 | 24030172 | 14941404 | 62.18 |
| HC4 | 23988688 | 15543063 | 64.79 |
| HC5 | 24022970 | 14436036 | 60.09 |
| LADA1 | 24084976 | 15601628 | 64.78 |
| LADA2 | 24065044 | 15340195 | 63.74 |
| LADA3 | 24101688 | 16577589 | 68.78 |
| LADA4 | 24066643 | 15133705 | 62.88 |
| LADA5 | 24081934 | 17194661 | 71.40 |

HC: heathy control; LADA: Latent autoimmune diabetes in adults; Mapped reads: The percentage of mapped reads (%)
